# Post-infectious Bronchiolitis Obliterans: HRCT, DECT, Pulmonary Scintigraphy Images, and Clinical Follow-up in Eight Children

**DOI:** 10.3389/fped.2020.622065

**Published:** 2020-12-18

**Authors:** I-Chen Chen, Jui-Sheng Hsu, Yu-Wen Chen, Yi-Ching Liu, Yen-Hsien Wu, Jong-Hau Hsu, Yi-Fang Cheng, Zen-Kong Dai

**Affiliations:** ^1^Department of Pediatrics, Kaohsiung Medical University Hospital, Kaohsiung, Taiwan; ^2^Department of Pediatrics, School of Medicine, College of Medicine, Kaohsiung Medical University, Kaohsiung, Taiwan; ^3^College of Medicine, Graduate Institute of Medicine, Kaohsiung Medical University, Kaohsiung, Taiwan; ^4^Department of Radiology, Kaohsiung Medical University Hospital, Kaohsiung, Taiwan; ^5^Department of Radiology, School of Medicine, College of Medicine, Kaohsiung Medical University, Kaohsiung, Taiwan; ^6^Department of Nuclear Medicine, Kaohsiung Medical University Hospital, Kaohsiung, Taiwan; ^7^Department of Nuclear Medicine, School of Post-Baccalaureate Medicine, College of Medicine, Kaohsiung Medical University, Kaohsiung, Taiwan

**Keywords:** perfusion, ventilation, dual-energy CT (DECT), pulmonary scintigraphy, children, high resolution computer tomography, post-infectious bronchiolitis obliterans

## Abstract

**Background:** Bronchiolitis obliterans (BO), first mentioned in 1901, is a severe and rare chronic lung disease in children. BO has various etiologies and the most common in children is post-infectious BO (PIBO). High resolution CT (HRCT) is an often-used image tool for the diagnosis of BO, and pulmonary scintigraphy is an alternative tool that can functionally evaluate BO. Recently, dual-energy computed tomography (DECT) have also been applied to BO for its accuracy and safety. Here we described the characteristics of HRCT, pulmonary scintigraphy, DECT, and the clinical profiles of patients with PIBO.

**Methods:** This is a retrospective and descriptive study. Data were collected from patients diagnosed with PIBO from 2014 to 2019 in the Pediatric Cardiopulmonary Outpatient Clinics of Kaohsiung Medical University Hospital. The diagnosis was based on clinical, chest X-ray, and HRCT findings. Clinical profile, radiological characteristics, and images of pulmonary scintigraphy were documented.

**Results:** Eight children (4 boys and 4 girls) were diagnosed with PIBO at a mean age of 25.8 months (range 15 to 41 months). Two of our patients developed pulmonary hypertension. The most common HRCT finding is mosaic pattern, where match ventilation/perfusion (V/Q) defects is a general feature in pulmonary scintigraphy. DECT pulmonary blood vasculature images revealed various degrees of decreased perfusion and is compatible with the decreased perfusion on pulmonary scintigraphy.

**Conclusion:** The therapeutic strategy of PIBO is still lacking of standardization. HRCT and V/Q scans are important image tools in diagnosis and follow-up of BO. DECT may be used in BO patients as it has no additional radiation exposure and add value on functional information of HRCT.

## Introduction

Bronchiolitis obliterans (BO) is an uncommon but severe lung disease (1). It is currently diagnosed according to a history of lower respiratory tract insults and persistent symptoms that do not respond well to the administration of systemic corticoids and bronchodilators for 2 weeks (2). Due to the chronic irreversible inflammatory process and limited treatment options for BO, it is important to make an early diagnosis and start treatment as soon as possible.

BO describes obliterative changes in the small airways that commonly occur in a variety of lung diseases, including lower respiratory infection, organ transplantation, connective tissue disease, toxic fume inhalation, chronic hypersensitivity pneumonia, aspiration, drugs and Stevens-Johnson syndrome (SJS) (2). However, post-infectious BO (PIBO) is the most common type in children (2). Histologically, two types of BO have been proposed: constrictive-type BO and proliferative-type BO (3). BO that develops during childhood is mainly the constrictive type, and it is characterized by peribronchiolar fibrosis with different degrees of lumen-narrowing (1). The severity of BO mainly depends on the degree of damage to normal tissue in the respiratory tract. However, due to the heterogeneous or patchy involvement of the disease, a lung biopsy has been reported to be non-diagnostic in up to one-third of patients (4). Moreover, due to concerns over the risk of invasive procedures in children, a lung biopsy is rarely performed in the diagnosis of BO (4). Currently, a confirmatory diagnosis is usually made according to typical clinical presentations, fixed airway obstruction on pulmonary function tests, and radiological findings (5). The most commonly used imaging methods to evaluate BO are conventional chest radiograph (CXR), high-resolution computed tomography (HRCT), and lung ventilation/perfusion (V/Q) scan (4, 6–8).

As the findings of BO on CXR are non-specific, HRCT is the most commonly used imaging tool for BO due to its high sensitivity and specificity, and because it can assess regional heterogeneity as well as the global severity of the lung. The typical findings of BO on HRCT include bronchial wall thickening, centrilobular opacities, central bronchiectasis, atelectasis, mucous plugging, and mosaic lung attenuation due to air trapping (6, 7, 9).

V/Q scans show a distinctive pattern of matched ventilation-perfusion defects and segmental, sub-segmental or lobar distribution in PIBO (10, 11). It provides an objective assessment of the distribution pattern of the lesions, and since they highlight more damaged broncho-pulmonary areas, it may also be considered to be an accurate diagnostic tool for BO (7). Furthermore, the degree of ventilation and perfusion abnormalities evaluated by V/Q scans have been associated with disease severity and may be used to predict the outcomes of patients with PIBO (12).

Dual-energy computed tomography (DECT) was first conceptualized in the 1970's (13–15). It enables the simultaneous evaluation of gray-scale vasculature with color-coded pulmonary blood vasculature (PBV) images, which represents parenchymal perfusion. DECT has been used to evaluate ventilation function after xenon inhalation, and this technique has been shown to provide more regional function information without additional radiation exposure in BO patients (4, 16). Therefore, the aim of this study was to investigate the diagnostic utility of HRCT, pulmonary scintigraphy, and DECT PBV images in our patients with PIBO. Moreover, we also discussed the initial clinical presentations and major treatment options for PIBO.

## Materials and Methods

This retrospective and descriptive study included patients with a diagnosis of PIBO who were followed up at the Pediatric Cardiopulmonary Outpatient Clinics of Kaohsiung Medical University Hospital and was approved by the Ethics in Research Committee of the institution where was conducted (KMUHIRB-SV(II)-20200063). The medical records of the enrolled children were reviewed retrospectively by Kaohsiung Medical University Hospital staff (KMUH) from January 2014 to December 2019. The diagnosis of PIBO was based on a typical clinical history followed by findings on CXR and thoracic HRCT that concurred with the diagnosis as follows: [1] history of acute and severe bronchiolitis/pneumonia; [2] recurrent cough, wheezing, respiratory distress after an acute event; [3] respiratory symptoms which were severe in disproportion to CXR findings; [4] mosaic pattern, air trapping or other typical patterns in HRCT; and [5] exclusion of other congenital heart diseases, immunodeficiency or V/Q scans and DECT were not available. Demographic information including age, sex, weight, onset of disease, clinical presentations, major treatment and cardiac echography reports were obtained.

### HRCT and DECT Pulmonary Blood Volume Fused Images

HRCT and DECT images were generated on a Dual Source CT (Siemens Somatom Definition) in dual energy mode at 140 and 80 kVp with 1-mm collimation. The images were retrieved from the hospital records and were read in a random order by two experienced radiologists. The CT scans were performed while the patients were stable with no acute respiratory tract infections. They were investigated under sedation if uncooperative. Heart rate, respiratory rate, and oxygen saturation levels were monitored continuously.

### Pulmonary ^99m^Tc-Diethylenetriamine Penta-Acetic Acid (DTPA) Radioaerosol Ventilation Scintigraphy

^99m^Tc-DTPA with an activity of ~370–900 MBq (10–25 mCi) was first put into a high-pressure oxygen jet nebulizer which produced radioaerosol particles at an oxygen flow rate of ~10 l/min (11). During inhalation, sealed oxygen masks were placed on the patient's face around the mouth region to minimize leakage of the radioaerosol into the surrounding environment. Anterior, posterior, and both lateral and posterior oblique views were acquired, with 250 Kcounts for each planar image using a gamma camera (Toshiba GCA 602A, LEGP collimator, Japan).

### Pulmonary ^99m^Tc-Macroaggregated Albumin Perfusion Scintigraphy

^99m^Tc-macroaggregated albumin (0.5–2.0 MBq/kg) was injected intravenously slowly during three to five respiratory cycles while the patients were in a supine position (11). Anterior, posterior, and both lateral and posterior oblique views were acquired with 500 Kcounts using the same gamma camera. Two experienced nuclear medicine physicians independently interpreted the V/Q scans. A matched defect was defined as ventilation and perfusion defects in the same location. A mismatched perfusion defect was defined as a perfusion defect not accompanied by a corresponding ventilation defect, and a mismatched ventilation defect was defined as a ventilation defect without a corresponding perfusion defect.

## Results

This retrospective and descriptive study included 10 patients with a diagnosis of PIBO who were followed up at our Pediatric Cardiopulmonary Outpatient Clinics. Of the 10 patients, two were excluded due to congenital heart disease and because a full image study could not be obtained. The remaining eight patients were enrolled in the study (four boys and four girls). The mean age at symptom onset was 25.8 months (range 15–41 months). At the time of diagnosis, most of the patients had cough, tachypnea or dyspnea, and wheezing or crackles on auscultation (Table 1).

**Table 1:** Clinical and imaging characteristics of the patients with BO.

**Case**	**Age (mo)**	**Sex**	**Etiology**	**Initial clinical presentation**	**PH**	**Major treatment**	**Main finding on HRCT**	**V/Q scan**	**Dual-energy CT**
1	41	F	*Mycoplasma pneumoniae*	Dyspnea, cough, fever, and crackle	N	(A) + (B) + (C) + (D)	Bronchiectasis, air-trapping, mosaic pattern, centrilobular nodules, atelectasis	Multiple V/Q match reduction	Decreased perfusion
2	20	M	*Mycoplasma pneumoniae*	Hemoptysis, fever, and dyspnea	Y	(B) + (C) + (D)+sildenafil	Mosaic pattern	Multiple V/Q match reduction	Decreased perfusion
3	17	F	NA	Dyspnea, cough, and crackle	Y	(A) + (B) + (C) + (D)+sildenafil	Mosaic pattern	V/Q match reduction in the left lung	Decreased perfusion
4	27	M	NA	Cough and dyspnea	N	(A) + (B) + (C)	Thickening	Bilateral reduced perfusion	Decreased perfusion
5	15	F	NA	Cough and dyspnea	N	(B) + (C) + (D)	Mosaic pattern, effusion, atelectasis,	Bilateral V/Q matched defect	Decreased perfusion
6	27	F	NA	Cough, dyspnea and wheezing	N	(B) + (C)	Mosaic pattern, thickening, atelectasis	Bilateral V/Q matched reduction	Decreased perfusion
7	26	M	*Mycoplasma pneumoniae*	Pneumomediastinum, dyspnea and wheezing	N	(A) + (B) + (C) + (D)	Mosaic pattern, atelectasis, pneumomediastinum	Bilateral V/Q matched reduction	Decreased perfusion
8	33	M	*Influenza*	Dyspnea on exertion, fever, crackle	N	(A) + (B) + (C)	Mosaic pattern, atelectasis	Decreased right pulmonary perfusion	Decreased perfusion

*mo, months; PH, pulmonary hypertension; N/A, Not available; V/Q, ventilation/perfusion; HRCT, high resolution computed tomography. (A) Long term azithromycin for BO; (B) short-acting β-agonist; (C) systemic corticosteroid; (D) montelukast*.

Three of our patients were serum Mycoplasma pneumoniae IgM positive and recognized as *Mycoplasma pneumoniae* related BO; one was positive rapid antigen tests for influenza and others are unknown etiology. The common initial clinical presentation including dyspnea, cough and fever. Interestingly, one case revealed recurrent pneumomediastinum and wheezing (case 7).

An echocardiogram was requested to rule out heart disease and indirectly evaluated pulmonary arterial pressures in all patients. The max velocity (Vmax) was determining by tricuspid regurgitation, and pulmonary hypertension was recognized while Vmax more than 2.8 m/s (17). Subsequently, six patients had normal systolic pulmonary artery pressure (SPAP), whereas two patients had higher SPAP, with a range from 35 to 46 mmHg of SPAP.

**Figure 1:**
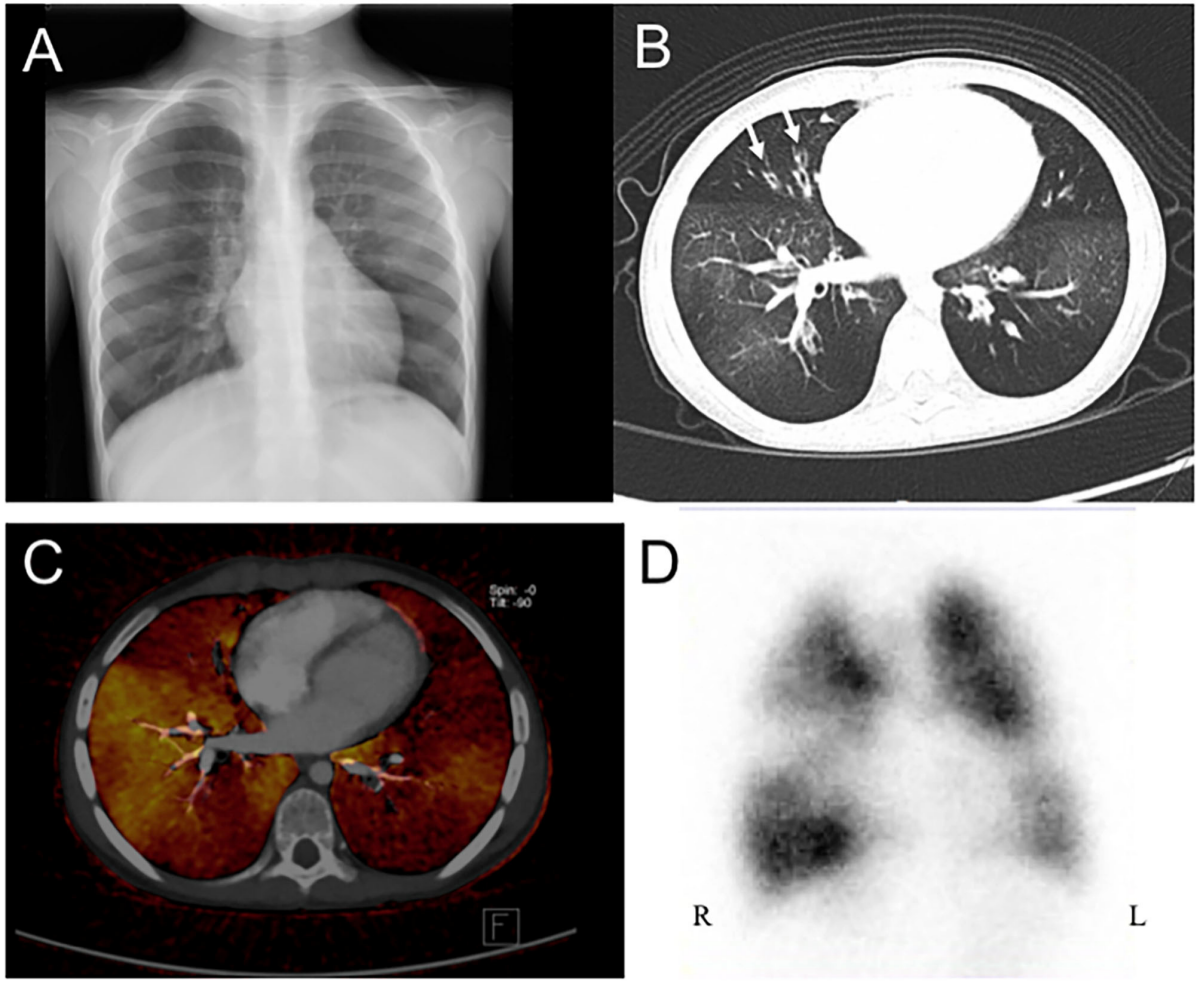
A 4 years old girl diagnosed with post-infectious with initial clinical symptoms of cough and dyspnea. **(A)** Chest X-ray revealed peribronchial thickening and emphysema. **(B)** Axial view of HRCT revealed mosaic pattern and bronchiectasis (arrow). **(C)** Axial view of DECT reveal regional decreased pulmonary blood vasculature, and **(D)** Perfusion scintigraphy showed reduction blood flow in bilateral lungs. HRCT, high resolution computed tomography; DECT, dual energy computer tomography; R, right side; L, left side.

**Figure 2:**
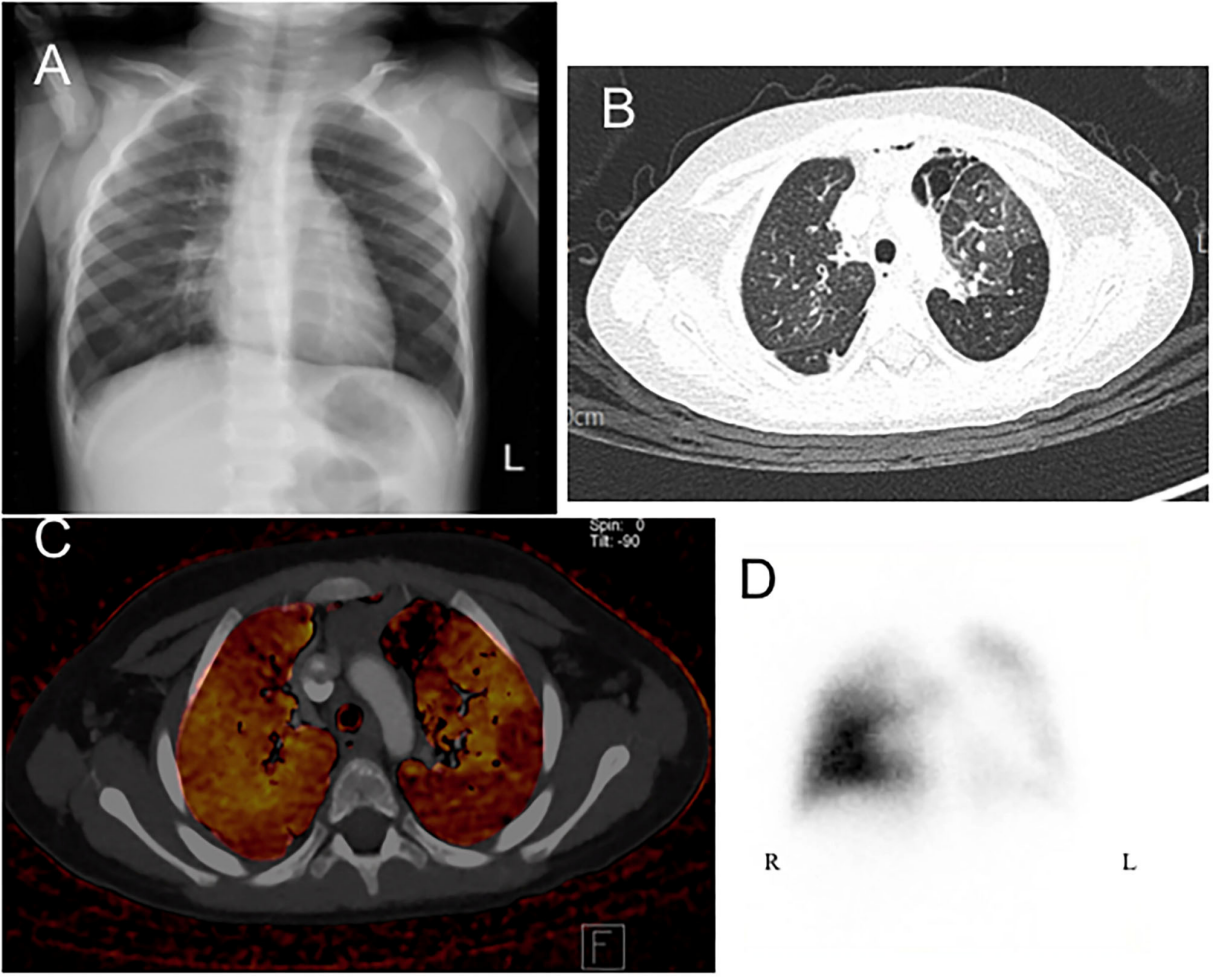
A 2-year-old boy presenting with tachypnea and dyspnea and receiving flexible bronchoscopy, which showed normal tracheobronchial appearance except much whitish sputum. **(A)** Chest X-ray revealed hyperinflation and attenuation of vascular marking in both fields. **(B)** Axial view of HRCT revealed mosaic pattern which are characterized by well-defined border, areas of decreased lung attenuation are associated with decreased pulmonary blood vasculature of DECT image **(C)**. **(D)** Perfusion scintigraphy revealed marked lobar defects in right upper, left lower, and left upper lung fields. HRCT, high resolution computed tomography; DECT, dual energy computer tomography; R, right side; L, left side.

All of the enrolled patients underwent HRCT, DECT, and pulmonary scintigraphy. In the main finding on HRCT, mosaic pattern is the most common feature (7/8) in our patients, followed by atelectasis (5/8). In lung pulmonary scintigraphy, two patients received only pulmonary perfusion scintigraphy, and the others received both ventilation and perfusion scintigraphy. In the 2 cases who only received perfusion scintigraphy, reduced pulmonary perfusion was noted, whereas unilateral or bilateral V/Q matched defects were present in the others. The DECT PBV image shows various degree of decreased perfusion, which is correlated to the decreased perfusion on pulmonary perfusion scan. Figures 1, 2 shows CXR, HRCT, perfusion scan, and reduced PBV images on DECT of case 1 and case 8.

Five patients (case 1, 4, 6, 7, 8) underwent spirometry during follow-up, and the others were younger than 7 years or uncooperative. The results showed one was normal (case 6); two were mixed type pulmonary dysfunction (case 1 and 7); one was airflow obstruction (case 4); and one was restrictive pulmonary dysfunction. Four (case 1, 4, 7, 8) had a decreased end-expiratory flow (MEF25 < 35%).

Treatment varied and was individualized. Systemic or inhaled corticosteroids were administered to all of the patients. The mode of steroid administration was chosen empirically according to the severity of the case. Inhaled bronchodilators (short-acting ß-2) were administered to all of the patients who had exacerbations of the respiratory condition and in those who responded to it clinically. Long-term azithromycin was given to 5 of the patients for immunomodulation. Montelukast was given to five of the patients to control an unstable chronic respiratory status. Moreover, sildenafil was also given to two patients with pulmonary hypertension (PH) secondary to BO and both showed marked improvement of oxygenation and estimated SPAP.

## Discussion

Various respiratory viruses or bacteria including adenovirus, respiratory syncytial virus (RSV), *Mycoplasma pneumoniae*, type B *Streptococcus, Legionella pneumophilia* and *Bordetella pertussis* have been investigated in relation to the development of PIBO (18–21). Viruses can be identified by polymerase chain reaction, detection of antibodies, rapid antigen test, or virus isolation in acute infections of the respiratory tract. In our study, some of the patients were only referred to our center weeks or months after the acute infection stage, and so it was not possible to verify the pathogens. Three of the 8 patients were *Mycoplasma pneumoniae* IgM-positive at the initial hospitalization and were diagnosed with *Mycoplasma pneumoniae-*related PIBO.

PIBO is more common in children, especially in those under 1 year old; however, age does not appear to be a risk factor for the development of PIBO (22–24). The common features of BO are tachypnea, wheezing, and hypoxemia persisting for at least 2 months after a causative event (5). In our patients, dyspnea, abnormal breath sounds on auscultation, and cough were the most common symptoms. To the best of our knowledge, only two articles have reported the prevalence of PH in BO. Nathan et al. reported that 42.3% of the lung transplant recipients in their study had an elevated pulmonary pressure (25), and Pate et al. reported that 3 of 4 patients (75%) were diagnosed with PH after the diagnosis of BO at a median of 91 days after hematopoietic stem cell transplantation (26). There was no such study in the aspect of PIBO and our study revealed that two of eight (25%) has PH and start the treatment with sildenafil once the diagnosis was made. Taken together, patients with PIBO should be regularly screened for PH due to its high prevalence. In addition, since hypoxemia presents in both BO and PH, hypoxemia in a patient with BO is typically due to worsening PH, and PH may also contribute to hypoxemia.

Steroid therapy has always been the central of BO treatment (2, 5, 7, 8). However, the side effects of the long-term systemic administration of glucocorticoids and inhaled corticosteroids have caused investigators to search for an alternative treatment for BO. Recently, macrolides have been proven to have anti-inflammatory and immunomodulatory effects, and they have begun to be used for post-transplantation BO. A comprehensive analysis in 2014 and a large-scale randomized clinical trial in 2015 confirmed that azithromycin can improve the lung function FEV1 and reduce mortality of patients with post-lung transplant BO syndrome (27, 28). The recommendation of macrolides to treat post-transplantation BO is Grade IA and Grade 2C for PIBO (29). A more recent study demonstrated that combination therapy with budesonide, montelukast and azithromycin could improve pulmonary function and respiratory symptoms in children with PIBO who were under 5 years of age compared to unconventional treatment (budesonide for nebulization intermittently, prednisone, montelukast and antibiotics if necessary) (30). In our study, 3 of the patients received azithromycin and montelukast and had clinical improvement.

The obliterative changes in BO include divergent histologic and radiologic findings, the ability to progress to additional compartments of the lung, and different clinical outcomes (31). CXR images are non-specific, and can sometimes be normal or present with air trapping, atelectasis, bronchial thickening, or a more unilateral hyperlucent lung, known as Swyer-James syndrome (22). Similar to other studies on HRCT in BO, we also found that a mosaic pattern/attenuation was the most typical feature, and others included atelectasis, peribronchial thickening, air-trapping, and bronchiectasis (2, 22, 32). The mosaic pattern may be caused by vascular shunt from hypo-ventilated areas to normal or hyper-ventilated areas with decreased perfusion due to vessel constriction caused by regional tissue hypoxia (22). To further understand the distribution of pulmonary blood flow, a V/Q scan can provide functional lung imaging to diagnose BO (33, 34). In accordance with our previous study (11), decreased V/Q-matched defects were the major finding of pulmonary scintigraphy in the patients with BO in the current study.

DECT produces accurate anatomic and functional images by manipulating the differences in the interactions of high- and low-energy photon spectra with the atomic factors of various materials and tissues to accurately discriminate the chemistry of tissues of the body. Xenon ventilation DECT can provide two key insights into lung physiology, i.e., regional perfusion and ventilation, and it has been actively investigated with regards to clinically relevant applications (4, 16, 35). This functional information provided by DECT is supplementary because high-resolution thoracic anatomy is entirely preserved on dual-energy thoracic CT. In addition, virtual non-contrast imaging can omit pre-contrast scanning. In this respect, DECT imaging is at least dose-neutral, which is a critical requirement for pediatric patients (35). To the best of our knowledge, no previous study has compared DECT PBV images to perfusion pulmonary scintigraphy in patients with PIBO. Although we could not perform xenon ventilation CT scans at our facility, the results are the first to show that DECT PBV images and pulmonary perfusion scans are compatible in BO patients. This result could suggest that DECT may be used in BO patients as it has no additional radiation exposure and provides regional pulmonary perfusion information as pulmonary perfusion scan.

## Conclusion

The therapeutic strategy and diagnostic tools of PIBO are lacking of standardization. HRCT and V/Q scans are important image tools in diagnosis and follow-up of BO, whereas DECT may be used in BO patients as it provides additional information on pulmonary vasculature. We suggested to gather DECT PBV when performing HRCT in patients with BO if available. Better understanding the image presentations and the feasible medication choice of PIBO will lead to better outcome for this lifelong respiratory disease.

## Data Availability Statement

The raw data supporting the conclusions of this article will be made available by the authors, without undue reservation.

## Ethics Statement

The studies involving human participants were reviewed and approved by KMUHIRB-SV(II)-20200063. Written informed consent from the participants' legal guardian/next of kin was not required to participate in this study in accordance with the national legislation and the institutional requirements.

## Author Contributions

I-CC and Z-KD: conceptualization. I-CC, Y-WC, Y-CL, Y-HW, and Z-KD: data collection. J-SH, J-HH, Y-CL, Y-HW, and Y-WC: validation. I-CC, J-HH, and Z-KD: formal analysis. J-SH and Y-WC: investigation. J-SH, Y-WC, and Z-KD: resources. I-CC: writing—original draft preparation. Z-KD: writing—review and editing. All authors have read and agreed to the published version of the manuscript.

## Conflict of Interest

The authors declare that the research was conducted in the absence of any commercial or financial relationships that could be construed as a potential conflict of interest.
